# A qualitative study of the reasons for delayed medical treatment in adolescents with depression based on the health ecology model

**DOI:** 10.3389/fpubh.2023.1124397

**Published:** 2023-07-25

**Authors:** Yuan Zhao, Yuling Li, Juan Zhao

**Affiliations:** ^1^School of Nursing, Shanxi Medical University, Taiyuan, Shanxi, China; ^2^Department of Nursing, The First Hospital of Shanxi Medical University, Taiyuan, Shanxi, China; ^3^Mental Health Center of the First Hospital of Shanxi Medical University, Taiyuan, Shanxi, China

**Keywords:** adolescents, depression, delayed access to care, health ecology model, qualitative study

## Abstract

**Background:**

Depression is a prevalent mental health issue among adolescents, and timely treatment can prevent it from worsening. However, many adolescent depressed patients delay seeking medical treatment. To clarify the reasons for delayed medical treatment in adolescent depressed patients and to provide reference to help adolescent depressed patients to seek medical treatment on time.

**Methods:**

From July 2022 to March 2023, a semi-structured interview was conducted using a purposive sampling method with adolescent depressed patients attending the Mental Health Center of the First Hospital of Shanxi Medical University. Based on inclusion and exclusion criteria, 22 adolescent depression inpatients with delayed access to medical care were finally included as the study population. This study applied the phenomenological research method in qualitative research and used the Colaizzi seven-step data analysis method to analyze and refine the interview data.

**Results:**

The study found that 22 adolescents with depression delayed medical care for 1–6 years, with a mean of 2.86 years. Six themes were identified as the reasons for delayed medical treatment: (1) inadequate levels of mental health literacy; (2) lack of disease-related knowledge and information; (3) negative emotional experiences; (4) negative attitudes toward health care; (5) inadequate medical insurance policy for psychotherapy; (6) unequal distribution of resources for mental health medical services.

**Conclusion:**

The phenomenon of delayed medical treatment for adolescent depression patients should not be ignored. Efforts should be made to explore the trajectory of delayed illness in adolescents with depression, improve patient symptom assessment, improve poor patient experience, promote positive patient behavior, and improve the current situation of delayed medical treatment for adolescent depression patients through the joint efforts of individuals, families, schools, and society.

## Introduction

Depression is a prevalent mental health issue affecting over 350 million people worldwide. It is the fourth most common disease globally and is predicted to be the number one disease burden by 2030, according to the World Health Organization (WHO) (1). Depression is prevalent among adolescents, and its incidence has increased in recent years, making adolescent depression a major public problem (2). Most often manifest as appetite and weight disturbances, fatigue, insomnia, and unexplained physical symptoms (such as musculoskeletal pain or headaches) (3). However, due to atypical symptoms and the adolescent Psychological transition period, patients and their families often ignore and delay medical treatment, leading to a diagnosis of major depression at the time of treatment, which not only increases the suffering of patients but also affects the prognosis of patients and increases the possibility of recurrence. Studies have shown that adolescent-onset depression can persist into adulthood, burdening individuals, families, and communities (4–6). A recent systematic evaluation found that adolescents with depression were approximately 2.5 times more likely to experience depression in adulthood than those without depression (5). In addition, adolescents with adolescent depression are at an increased risk for fatal suicide attempts and completed fatal suicide attempts in adulthood (7).

The health ecological model (HEM) (8) suggests that personal attributes, family factors, and social support influence an individual’s health. The model is based on an inside-out approach that includes innate personality traits, individual psychology, behavior, lifestyle, interpersonal relationships in the family community, work and living conditions, and policy environment. Delayed access to care (9) refers to the time between the first detection of symptoms and the patient’s first visit to a health care facility, with most studies defining ≥3 months as delayed access to care. At the same time, there are many studies on delayed access to care for physical diseases, including but not limited to chronic obstructive pulmonary disease, cancer, and coronary heart disease (9–12). There are few national studies on delayed health care seeking among adolescents with depression.

Compared to adults, the first episodes of depression usually occur in adolescents (3). Depressive symptoms in adolescents are more insidious and have a higher recurrence rate. Timely access to treatment can prevent further deterioration of depression and reduce the occurrence of fatal suicide attempt behavior in adolescents (13). Therefore, this study aims to investigate the reasons for delayed access to treatment in adolescents with depression through a semi-structured interview method and provide a reference for guiding them to access treatment times and improve treatment outcomes.

## Methods

### Participants

Adolescent depression inpatients attending the Mental Health Department of the First Hospital of Shanxi Medical University from July 2022 to March 2023 were selected using purposive sampling. The sample size was determined because no new themes would emerge, and the information was saturated. Inclusion criteria for the study subjects: (1) meeting the diagnostic criteria of depression in the International Classification of Diseases [ICD-10 (14)]; (2) age 10–19 years (1); (3) time from the onset of symptoms to the first visit to the doctor was more than 3 months; (4) having some communication skills; (5) voluntarily participating in this study with the consent of their guardians and signing the informed consent form. Exclusion criteria: (1) high fatal suicide attempt tendency; (2) major physical illness or other psychiatric disorders such as schizophrenia.

This study finally included 22 hospitalized adolescents with depression.

The ethics committee of the First Hospital of Shanxi Medical University approved this study (approval number: 2022, ethics number K-139). All participants were informed of the study’s purpose, methods, and privacy and signed informed consent forms.

### Procedures

Data were collected using a semi-structured interview method. An initial interview outline was developed based on a literature review of health ecology models (9, 15–17). The initial outline was revised by consulting our psychotherapist, mental health physician, and mental health nurse specialist. Before the formal interview, two adolescents with depression who met the inclusion and exclusion criteria were selected for pre-interview to supplement and improve the interview outline. The interview’s contents were as follows: (1) When did you first experience depressive symptoms? (2) Please talk about your feelings and thoughts at that time. (3) Did you talk about it with your family and friends then, and what was their reaction? (4) What do you think were the reasons that affected your need for timely help? Are there any other reasons? (5) Could you please tell us what you know about depression? (6) What support would you like to receive from your family, society, and medical personnel? The interview location selected the psychotherapy room in the ward. Explain the purpose and significance of the study to the respondents before the formal interview. The interview was recorded with their consent, and numbers replaced their real names. The researcher remained neutral during the interview, positively responded to the interviewees’ answers, and encouraged them to express their true thoughts. In addition to recording the verbal information of the interviewees, the expressions and movements were closely observed and recorded during the interview. Each interview ranged from 20 to 40 min. A circular research model was used to analyze the data simultaneously with the data collection. The extracted prototype themes guide the subsequent data collection, and the subsequent data collection is also a test of the prototype themes. This cycle continues until the data meaning is saturated.

### Data analysis

The recordings were converted into textual content within 24 h after the interviews, and the recorded information was repeatedly analyzed and labeled using the Colaizzi analysis method (18), naming the interviewees with P1 to P22. The collated information was verified with the respondents on time to ensure the authenticity of the data. To improve the reliability of this study, using the method of triangular mutual evidence, in which all information was coded back-to-back by two researchers, who questioned and supplemented each other’s interpretation of the material, summarized and extracted meaningful statements, developed themes, and repeatedly discussed with experts in the field to finalize the themes to reduce bias. The final results were returned to the patients for verification and validation, and corresponding modifications and refinements were made based on the feedback results to ensure the authenticity of the results.

### Quality control

The researcher systematically studied qualitative research in advance, understood qualitative research methods and interview techniques, and conducted pre-interviews before the formal interviews to ensure the feasibility of the study.

## Results

Twenty-two patients were included, including 5 males and 17 females, aged 13–19 years, with a mean of 16.32 years, and with a delay in medical care of 1–6 years, with a mean of 2.86 years. Specific information is shown in Table 1.

**Table 1:** Basic information on the participant demographics.

Number	Gender	Age (years)	Education	Age of first occurrence (years)	Duration of illness (years)	Place of residence	Primary caregiver	Parental marital status
P1	Female	13	Junior High School	10	3	Xinzhou	Parents	Married
P2	Male	16	High School	15	1	Xinzhou	Parents	Married
P3	Female	14	Junior High School	12	2	Qingxu	Parents	Married
P4	Female	16	Junior High School	13	3	Taiyuan	Grandparents	Divorced
P5	Female	17	High School	15	2	Taiyuan	Parents	Married
P6	Female	19	Undergraduate	15	4	Linfen	Parents	Married
P7	Female	16	High School	14	2	Changzhi	Parents	Married
P8	Male	16	High School	14	2	Yangquan	Parents	Married
P9	Female	17	High School	14	3	Shuozhou	Parents	Married
P10	Female	18	College	13	5	Xinzhou	Parents	Married
P11	Female	19	College	15	4	Xinzhou	Parents	Married
P12	Female	14	Junior high school	11	3	Taiyuan	Parents	Married
P13	Female	15	High School	13	2	Xinzhou	Parents	Married
P14	Female	17	High School	12	5	Jinzhong	Parents	Married
P15	Male	13	Junior high school	11	2	Lvliang	Parents	Married
P16	Male	18	High School	12	6	Taiyuan	Parents	Married
P17	Female	16	High School	14	2	Linfen	Parents	Married
P18	Female	17	High School	12	5	Jinzhong	Parents	Married
P19	Male	19	College	18	1	Shuozhou	Parents	Married
P20	Female	16	High School	13	3	Xinzhou	Parents	Married
P21	Female	19	College	18	1	Xinzhou	Parents	Married
P22	Female	14	Junior high school	12	2	Taiyuan	Parents	Married

**Figure 1:**
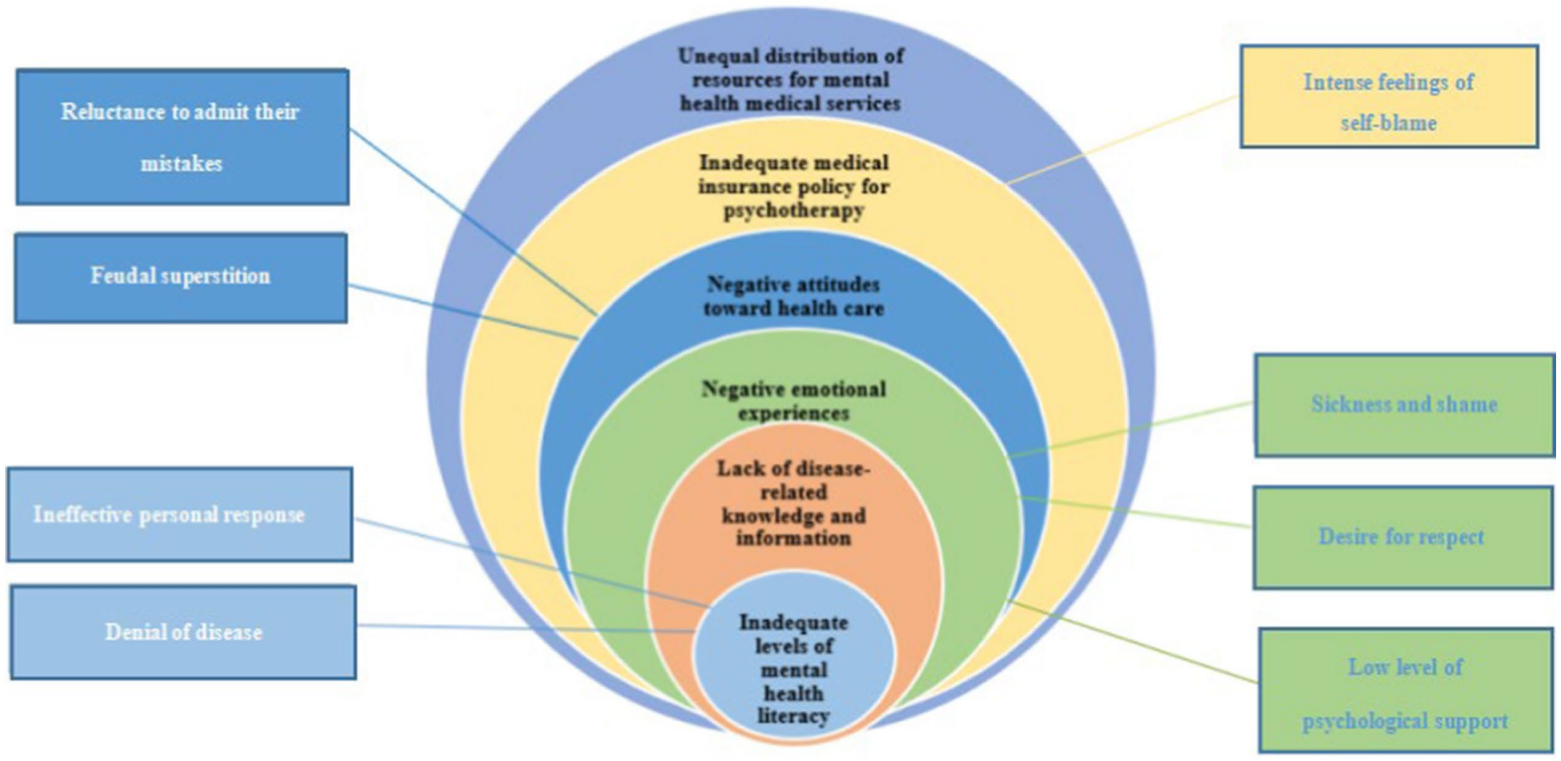
Health ecology model of the causes of delayed access to care in adolescents with depression.

A total of 22 people were interviewed in this study. A total of 6 themes and 8 Subthemes were eventually extracted based on the health ecology model, as shown in Figure 1.

### Theme 1: inadequate levels of mental health literacy

Mental health literacy (19) refers to an individual’s knowledge and beliefs about identifying, managing, and preventing mental disorders. The longer delay in seeking medical care for adolescents with depression may be related to the lack of their mental health management skills. At the same time, depression is hidden and imperceptible.

#### Subtheme 1: ineffective personal response

Most participants expressed a lack of ability to cope with depressed emotions and often felt helpless and hopeless. Parents fail to take themselves to a professional doctor in time and seek professional help only after others make suggestions. P1: “I have been putting it off for about 3 years since I discovered I had the disease.” My teacher told me that I could no longer delay and that it would be harder to treat the disease, so I decided to come here this time. P3 said, “I was happy for a long time in the beginning and depressed for a short time, so I did not take it seriously.”

#### Subtheme 2: denial of disease

Many participants were reluctant to admit that they had psychological problems and considered them normal. P5 “I do not think I’m sick, even now in the hospital. Is this a disease?” P11: “I do not think I already have this mental illness. I probably cannot sleep at night.”

### Theme 2: lack of disease-related knowledge and information

Lack of depression-related knowledge among patients and caregivers contributed to the delay in seeking medical care, and parents’ misattribution of depressive symptoms was an important factor in seeking medical care, attributing it to normal academic stress, normal falling out among classmates, and normal Psychological transition period of children. P1: “My parents do not even listen to me and think I belong to the Psychological transition period of adolescence.” P3: “My grandmother always thinks that little dolls are not that sick, it is inevitable that little dolls stumble and trip with each other, and she thinks that it is just a conflict between classmates and it will be fine after 2 days.” P11: “I would check Baidu, and many of the side effects turned me off.”

### Theme 3: negative emotional experiences

#### Subtheme 1: sickness and shame

Stigma about adolescent psychological problems is important in prolonging medical visits (20). Because of stigma, some parents are not willing to mention it to others, worried that it will affect their children’s study and life. P5: “After I cut my wrists, my grandma did not take me to the hospital. She wrapped it in her home. My grandma said not to let others know that you cut your wrists.” P12: “My family said not to tell people about the condition.” P17: “Many people do not understand, and I feel that having this disease is a defect.” P19: “I am worried about being looked at differently by others.”

#### Subtheme 2: desire for respect

Some participants said that parents cared too much about their image and reputation and were worried about being talked about by colleagues. P1: “My father works in the Education Bureau and is dignified in the unit, and my father is worried that others will talk about his family’s children who have a mental illness.”

#### Subtheme 3: low level of psychological support

Many participants expressed a lack of psychological care from their parents. P10: “My mom said I’m not sick, everyone is depressed, and I’m nothing.” P13: “My mom sometimes says, buy you whatever you want. What else do you want?” P14: “When I was a child, my parents worked in other places and got along with less, easy to neglect me.” p20: “Parents’ career reasons, work is relatively busy, we communicate less.”

### Theme 4: negative attitudes toward health care

The patient’s and family’s attitude toward the disease can influence the medical treatment-seeking behavior. In the communication with the patients, it found that most of the children themselves proposed hospitalization, and the families would often avoid and deny the truth about their children’s illnesses. They are reluctant to acknowledge the mental illness of the child patient, thus causing a delay in the outcome of the medical consultation.

#### Subtheme 1: reluctance to admit their mistakes

Participants indicated that their parents were unwilling and afraid to admit their problems. P2: “My parents did not think their education methods caused my depression. There is a fear of admitting their mistakes and unwilling to make a change.” P6: “My father went to a family therapy course. He thought he had no problem with his education method and was unwilling to admit that he was wrong.”

#### Subtheme 2: feudal superstition

Participants said that their parents’ traditional superstition was deeply rooted and could not take a scientific approach to seek help. P2: “My parents would rather spend 2,000 yuan to pray to God than 100 yuan for psychotherapy.” P3: “My mother heard the old man say that this kind of problem can invite the Buddha home, so my parents invited the Buddha home and put it in the living room.” P7: “When I was in a bad mood, my parents felt that the child’s body was a feng shui problem, which forced me to drink ash water (burning ash into water).” P8: “My mother did not know where to ask, returned home to give me to drink water (burning ash into water).” P9: “I was having problems, and they did not take me to the doctor but first went to Wutai Mountain to pray to the gods and Buddha.”

### Theme 5: inadequate medical insurance policy for psychotherapy

The cost of psychotherapy is high and not included in the health insurance policy, so all costs are borne by oneself. The family’s financial situation can not guarantee the course of treatment. P6: “I heard that psychotherapy costs hundreds of yuan at a time, and you must go at least once a week. It’s too expensive.”

#### Subtheme 1: intense feelings of self-blame

Most adolescents see themselves as a burden to the family, and the cost of psychotherapy can make the family more financially constrained. P4: “I see the bedside electronic billing only a few days just so much money, I do not want to treat.” P15: “My family’s economic condition is general, the cost that long-term treatment cost produces is too much, the feeling oneself is cumbersome.” P17: “My family’s financial condition is relatively poor, and I feel I will add a burden to my family.”

### Theme 6: unequal distribution of resources for mental health medical services

On the one hand, grassroots medical personnel needs to gain the relevant knowledge and technology to diagnose the disease correctly, and often misdiagnosis leads to delays in seeking medical treatment. On the other hand, it is far from the big hospitals, and the beds in the big hospitals are tight. P8: “My home was in the county seat. Before also went to the local hospital to see and was prescribed a little medicine. Eating also had no effect.” P9: “I came to tell me that the hospital beds were tight and there were no empty beds yet, so I was asked to leave my phone number and contact me if I had a bed.” P3: “My mother was afraid of delaying my study and said to take me to a big hospital to see a doctor after winter and summer vacation.”

## Discussion

### Delayed access to care for adolescents with depression cannot be ignored

The results of this study have clear, practical implications for improving delayed access to care for adolescents with depression. Consistent with previous studies (21), a concerted effort from the individual patient, family, and society is needed. The World Health Organization reported that depression is not only the leading cause of adolescent morbidity but has become the third leading cause of disability-adjusted life years lost among adolescents (22, 23). Adolescent depression is not only characterized by high rates of suicide and relapse but also harms their schooling, life, and interpersonal communication (24, 25). In this study, 22 adolescents with depression were interviewed, and the results showed that the average delay duration was 2.86 years, and the longest had been 6 years. Radez et al. (21) showed that clarifying the reasons for not seeking professional help is crucial to addressing adolescent mental disorders’ high prevalence and low treatment rates. Therefore, a detailed understanding of the causes of delayed treatment and illness trajectories is essential to provide interventions for prevention, screening, timely diagnosis, and treatment to help adolescents with depression.

### Delayed disease trajectory in adolescents with depression who seek medical care

The healthcare-seeking behavior of adolescents with depression is influenced by multiple factors such as illness, individual, and family, and the process is not a single linear one. Studies show (26) that adolescent depression is a progressive disorder that may have long-term adverse effects on adolescents if not intervened on time. When treating depression, attention should be paid to the trajectory of the disease, understanding its progression, and adjusting the treatment plan according to the specific situation. In this study, we conducted semi-structured interviews with adolescent depression patients to explore the disease trajectory from the discovery of symptom problems to seeking medical care and analyzing and summarizing the common reasons for the occurrence of delays to explore countermeasures.

### Improve patient symptom assessment

Respondents in this study reported inadequate mental health literacy and a lack of knowledge related to their illness, leading to delayed assessments of symptoms and hindering early diagnosis. To address this issue, mental health literacy must be improved. As one of the most fundamental, cost-effective, and efficient measures to improve mental health, enhancing mental health literacy can effectively reduce delays in treatment for depression, which is essential to reduce its occurrence (27). Disseminating knowledge in an easy-to-understand manner using popular science videos, science brochures, and public service announcements for depression can significantly boost public mental health literacy. A high level of mental health literacy enables early identification of mental illness, reduces stigma, and ensures timely and practical support and treatment (28). Li et al. (29) developed a mental health literacy assessment scale validated in 3,826 medical students, including four dimensions of knowledge, identification, attitude, and behavior, with 36 entries proving effective in assessing college students’ mental health literacy level.

### Improving poor patient experience

The study findings suggest that adolescents’ negative emotional experiences and parental attitudes toward medical care severely impact access to care. Thus, creating public awareness regarding the prevalence of psychological problems is essential in reducing the stigma surrounding patients and their families, ultimately leading to a comfortable response to treatment. Self-stigma in adolescent depressive disorders mediates the effect of depressive mood levels on the willingness to seek help, with a higher degree of self-stigma presenting a lower likelihood of seeking help among adolescents. Therefore, one reason for the delay in seeking medical care is the reluctance to seek professional help due to increased self-stigma (30). Radez et al. (21) have also shown that public stigma, embarrassment surrounding mental health problems, and a lack of knowledge and availability of mental health care are barriers to seeking help. The Ministry of Education has taken a positive step by extensively disseminating knowledge about depression through media outlets such as film and television. Mental health education can increase public knowledge about depressive disorders and improve the mental health literacy of adolescents about these disorders (31).

### Promote positive patient behavior

A combination of medication and psychotherapy is the primary treatment for depression (25). The study showed that psychotherapy is more expensive and financially burdens families, affecting patients’ access to medical care. This is consistent with Li Hongjuan’s study (32). At present, Jiangsu and other places have included “psychotherapy” in the scope of medical insurance payment, and the state further increases the medical insurance coverage of psychotherapy to achieve full coverage as soon as possible. In addition, we should pay attention to the patient’s medical experience and the psychological sensitivity of adolescents, strengthen humanistic care in consultation and treatment, and pay attention to communication methods to improve the medical experience. Primary mental health is the substrate of our health prevention and treatment system and health service network (33). This study also showed that the unequal distribution of resources for mental health medical services led to the inability of primary institutions to make correct diagnoses of illnesses, consistent with the study of Zhong et al. (10). They are establishing a grassroots mental health workforce and driving the development of grassroots mental health through mentoring at the grassroots level, providing opportunities for further training, and remote online learning under the medical consortium model. The Ministry of Education (34), in its proposal on further implementation of measures to prevent and treat depression in adolescents, also pointed out that medical and health institutions at all levels should standardize and continuously conduct training on depression prevention and treatment and other related knowledge. Training for physicians in non-psychiatric hospitals should be increased to improve their ability to identify depression.

## Limitation

This study only explored the reasons for delayed medical care from the perspective of adolescent depressed patients without interviewing the patients’ caregivers to understand the thoughts of the caregivers. Also, the interviewees were from one hospital, and the sample size was small.

## Conclusion

This study conducted semi-structured interviews with 22 adolescents with depression based on the Health Ecology Model to explore in depth the reasons influencing their delay in seeking medical care, and a total of six themes were extracted to provide a reference for improving the phenomenon of delayed access to care for adolescent depressed patients.

Future studies could, on the one hand, understand the reasons for delayed medical care for adolescent depressed patients from the caregiver’s perspective and, on the other hand, develop quantitative self-reported indicators to clarify the influencing factors while conducting a large sample and multicenter study.

## Data availability statement

The original contributions presented in the study are included in the article/supplementary material, further inquiries can be directed to the corresponding author.

## Ethics statement

Written informed consent was obtained from the individual(s), and minor(s)’ legal guardian/next of kin, for the publication of any potentially identifiable images or data included in this article.

## Author contributions

YL and JZ designed the study and writing—review and revising. YZ and YL: data curation. YZ and JZ: formal analysis. YZ: writing—original draft. JZ obtained the funding. All authors contributed to the article and approved the submitted version.

## Funding

The study was funded by the Science and Technology Department of Shanxi Province (No. 202103021223435).

## Conflict of interest

The authors declare that the research was conducted in the absence of any commercial or financial relationships that could be construed as a potential conflict of interest.

## Publisher’s note

All claims expressed in this article are solely those of the authors and do not necessarily represent those of their affiliated organizations, or those of the publisher, the editors and the reviewers. Any product that may be evaluated in this article, or claim that may be made by its manufacturer, is not guaranteed or endorsed by the publisher.
